# Physico-Mechanical Properties of 3D-Printed Filament Materials for Mouthguard Manufacturing

**DOI:** 10.3390/polym17162190

**Published:** 2025-08-10

**Authors:** Maciej Trzaskowski, Gen Tanabe, Hiroshi Churei, Toshiaki Ueno, Michał Ziętala, Bartłomiej Wysocki, Judyta Sienkiewicz, Agata Szczesio-Włodarczyk, Jerzy Sokołowski, Ewa Czochrowska, Małgorzata Zadurska, Elżbieta Mierzwińska-Nastalska, Jolanta Kostrzewa-Janicka, Katarzyna Mańka-Malara

**Affiliations:** 1 Department of Prosthodontics, Medical University of Warsaw, 02-091 Warsaw, Poland; maciej.trzaskowski@wum.edu.pl (M.T.); elzbieta.mierzwinska-nastalska@wum.edu.pl (E.M.-N.); jolanta.kostrzewa-janicka@wum.edu.pl (J.K.-J.); 2 Department of Sport Dentistry, School of Dentistry, Meikai University, Sakado 350-0248, Japan; genspmd@tmd.ac.jp (G.T.); tueno@dent.meikai.ac.jp (T.U.); 3 Department of Masticatory Function and Health Science, Institute of Science, Tokyo 113-8510, Japan; chu.spmd@tmd.ac.jp; 4 Multidisciplinary Research Center, Cardinal Stefan Wyszynski University in Warsaw, 05-092 Dziekanów Leśny, Poland; m.zietala@uksw.edu.pl (M.Z.);; 5 Faculty of Mechatronics, Armament and Aerospace, Military University of Technology, 00-908 Warsaw, Poland; judyta.sienkiewicz@wat.edu.pl; 6 University Laboratory of Materials Research, Medical University of Lodz, 90-419 Lodz, Poland; agata.szczesio@umed.lodz.pl; 7 Department of General Dentistry, Medical University of Lodz, 90-419 Lodz, Poland; jerzy.sokolowski@umed.lodz.pl; 8 Department of Orthodontics, Medical University of Warsaw, 02-091 Warsaw, Poland; ewa.czochrowska@wum.edu.pl (E.C.); malgorzata.zadurska@wum.edu.pl (M.Z.)

**Keywords:** dental materials, sport dentistry, dental trauma, additive manufacturing, material extrusion, FDM printing, 3D printing, CAD/CAM, polymers, dynamic properties, abrasion resistance

## Abstract

Mouthguards are recommended for all sports that may cause injuries to the head and oral cavity. Custom mouthguards, made conventionally in the thermoforming process from ethylene vinyl acetate (EVA), face challenges with thinning at the incisor area during the process. In contrast, additive manufacturing (AM) processes enable the precise reproduction of the dimensions specified in a computer-aided design (CAD) model. The potential use of filament extrusion materials in the fabrication of custom mouthguards has not yet been explored in comparative studies. Our research aimed to compare five commercially available filaments for the material extrusion (MEX) also known as fused deposition modelling (FDM) of custom mouthguards using a desktop 3D printer. Samples made using Copper 3D PLActive, Spectrum Medical ABS, Braskem Bio EVA, DSM Arnitel ID 2045, and NinjaFlex were compared to EVA Erkoflex, which served as a control sample. The samples underwent tests for ultimate tensile strength (UTS), split Hopkinson pressure bar (SHPB) performance, drop-ball impact, abrasion resistance, absorption, and solubility. The results showed that Copper 3D PLActive and Spectrum Medical ABS had the highest tensile strength. DSM Arnitel ID 2045 had the highest dynamic property performance, measured with the SHPB and drop-ball tests. On the other hand, NinjaFlex exhibited the lowest abrasion resistance and the highest absorption and solubility. DSM Arnitel ID 2045’s absorption and solubility levels were comparable to those of EVA, but had significantly lower abrasion resistance. Ultimately, DSM Arnitel ID 2045 is recommended as the best filament for 3D-printing mouthguards. The properties of this biocompatible material ensure high-impact energy absorption while maintaining low fluid sorption and solubility, supporting its safe intra-oral application for mouthguard fabrication. However, its low abrasion resistance indicated that mouthguards made from this material may need to be replaced more frequently.

## 1. Introduction

The use of mouthguards is recommended to reduce the incidence and severity of sport-related oral injuries [1,2,3,4]. The use of elastic polymeric materials in their construction enables effective absorption and redistribution of impact energy [5,6,7,8]. The most thoroughly tested material for intra-oral protective splints is ethylene vinyl acetate (EVA) [5,6,7,9,10,11]. It is applied in thermoformed mouthguards, which currently are the gold standard in protective equipment. However, due to problems with thinning and inconsistent thickness, new solutions for protective splint formation should be considered [9,10,11,12,13]. Alternative techniques such as pressure injection and flasking offered high predictability in dimensions together with adequate retention and user comfort, yet failed to achieve widespread popularity [14,15,16]. Although the mechanical properties of the materials were highly favourable, the complex laboratory procedures requiring an experienced dental technician, and the limited availability of materials posed significant challenges. Additionally, in the case of injection moulding, the need for expensive supplementary equipment further hinders the widespread adoption of these manufacturing methods. Nowadays, three-dimensional (3D) printing is a viable option for manufacturing dental appliances [17,18,19]. This method provides high precision and the possibility to create complex geometries and multi-material structures. Furthermore, optimal occlusal contacts can be designed digitally to ensure stability and effective redistribution of impact energy [20,21,22]. Fully balanced occlusion in a protective splint reduces displacement of the mouthguard during the impact and lowers the forces transmitted to stomatognathic system structures.

Research on optimal materials and printing techniques for mouthguards is ongoing. A material intended for this purpose must have high-impact energy absorption, be resistant to the oral cavity environment and biocompatible, and provide predictable dimensions of the final protective appliance. Additionally, due to the need for frequent replacement, cost-effectiveness is an important factor from the user’s perspective. Researchers have evaluated available biocompatible materials, but to date no solution has been definitively recommended for the fabrication of digital mouthguards. Among the materials considered for digital manufacturing of such appliances were resin materials, extrusion-based filaments (fused deposition modelling (FDM) printing), and milled polyetheretherketone (PEEK) [8,22,23,24,25,26]. The high precision of elastic, polymeric, 3D-printed resins and their favourable properties have already been described [8,23,24,25]. The laser curing of subsequent layers ensures high precision in the final prints. Additionally, there is a possibility of using not only elastic light-cured materials, such as resins for indirect bonding of orthodontic brackets (IDB—indirect bonding resin), but also stiff materials for hard inserts [25,26,27]. The application of a harder structurally modified layer beneath the flexible outer material of the mouthguard may increase its mechanical durability without requiring direct contact between the oral cavity tissues and the rigid material during use. However, light-curing technologies—stereolithography (SLA), digital light processing (DLP), and polyjet printing—require costly materials and/or time-consuming ultraviolet (UV) light and chemical (isopropanol) post-processing. On the contrary, FDM printers are relatively easy to use, while filament materials are less expensive than resins [28]. The molten thermoplastic filament is extruded directly through the nozzle onto the working table layer by layer, creating a final three-dimensional object [29,30]. The high variety of materials that can be 3D-printed and the possibility of multi-material 3D printing, either by changing the filament during printing or using multi-extruder printers, allows for the creation of appliances of different mechanical properties, internal structure, and high personalisation while maintaining relatively low costs of manufacturing. Beyond commercially available materials, emerging modifications of filament-based polymers, such as PETG–EVA (polyethylene terephthalate glycol–ethylene-vinyl acetate) blends, with superior shape recovery and increased toughness appear promising and may in the future expand the range of clinically applicable options [31].

This research aimed to compare the mechanical properties of different filaments that could potentially be used in mouthguard manufacturing using extrusion-based additive manufacturing. Although manufacturers mention the possibility of using filament materials for protective splint fabrication, to date there has been no comparison in the literature of the material properties of filament materials that may be suitable for such intra-oral application. The distinctive feature of this study was evaluating the suitability of commercially available materials that enabled the assessment of whether the current state of filament-based 3D-printing technology is adequate for the broadly available digital manufacturing of intra-oral mouthguards. This is the first study to compare commonly used thermoformed EVA sheets, which currently represent the gold standard. Comparison of mechanical properties only in the context of the intended clinical application allows for the selection of the most suitable material. Additionally, the inclusion of materials of increased stiffness creates the possibility for further development of intra-oral mouthguard structure modifications, but now specifically in the field of material extrusion (MEX) printing. In this study, we undertook a comparative evaluation of physico-mechanical properties using tensile strength, split Hopkinson pressure bar, drop-ball impact, abrasion resistance, absorption, and solubility tests. Employing various testing methods to evaluate impact energy absorption enabled broader comparability of the obtained results with future studies.

## 2. Materials and Methods

### 2.1. Materials and Sample Preparation

Five commercially available filaments for extrusion-based 3D printing were selected: PLActive (Copper 3D, Santiago, Chile), Spectrum Medical ABS (Spectrum Filaments, Pęcice, Poland), Braskem Bio EVA (Braskem, Philadelphia, PA, USA), DSM Arnitel ID 2045 TPC (DSM, Exton, PA, USA), and NinjaFlex TPU (Ninjatek, Manheim, PA, USA) (Table 1). All materials had a diameter of 1.75 mm. Additionally, for control samples, ethylene vinyl acetate plates produced by Erkoflex (Erkodent, Pfalzgrafenweiler, Germany) were used. All specimens were tested at room temperature (23 °C).

Printed samples for each test were designed in Netfabb 2024 Premium (Autodesk, San Francisco, CA, USA) software. The orientation of all samples was adjusted to align parallel to the build platform within the slicing software, thereby eliminating the requirement for support structures. STL files were subsequently exported for fabrication. The CAD models were imported into PrusaSlicer software (2.6.0 version), where the primary printing parameters were defined according to Table 1 and saved in a format compatible with the Prusa MK3+ printer (Prusa, Prague, Czech Republic). All samples were fabricated on the Prusa MK3+ 3D printer using a standard grid-shaped infill of 80%. This infill pattern is the simplest and most widely used option in fused deposition modelling (FDM) printing, providing a balance between fast fabrication, straightforward nozzle trajectories, and satisfactory mechanical stiffness of the printed parts. Furthermore, the grid-shaped infill is the default setting in PrusaSlicer for common thermoplastic materials, including PLA, ABS, TPU, and other elastic or rubber-like filaments. Since the mouthguard was intended to be as solid as possible, a high infill density was selected. However, using densities above 80% is generally not recommended in FDM printing, as it significantly increases printing time without substantially improving the mechanical properties of the final part [32]. In fact, infill densities of 90% have been reported to cause earlier plastic deformation of the material under lower stress levels compared to 70% infill, while providing almost identical tensile strength [33].

Most extrusion parameters were initially set according to the recommendations provided by the filament manufacturers, which specify parameter ranges for different FDM printers and materials. The material profiles developed by 3D printer manufacturers are particularly useful, as they define optimal printing conditions for specific filaments. In this study, the predefined filament profiles supplied within PrusaSlicer by Prusa—the manufacturer of the MK3+ desktop 3D printer—were used. These profiles include parameters such as printing speed, temperature, and infill type, optimized for each material type. The main objective of this research was to assess the feasibility of using commercially available filaments in material extrusion (MEX) technology for the fabrication of custom mouthguards.

Nevertheless, slight adjustments to the default printing parameters were necessary after the first test prints to ensure proper fabrication of the samples, mainly due to their specific geometry and scale. All tested materials initially showed insufficient bed adhesion when printed using the default material profile settings from the PrusaSlicer library. To mitigate this issue and avoid detachment of the print—or in the case of flexible materials, to prevent the “elephant’s foot” defect caused by spreading of the first printed layer—the first two layers were printed with modified settings. Specifically, cooling fans were disabled during the initial layers for all materials to avoid rapid cooling, which can cause shrinkage and poor adhesion to the print bed. Additionally, for all elastic filaments (EVA, Arnitel, and NinjaFlex), retraction was disabled to prevent filament clogging in the nozzle. In summary, minor modifications to the default and manufacturer-recommended printing parameters were necessary to adapt the material extrusion process for the tested filaments on the Prusa MK3+ printer.

Reference samples were cut from EVA plates (Erkoflex, Erkodent, Pfalzgrafenweiler, Germany) using a cutting tool in the form of a circular knife mounted on a powered rotary holder, which was vertically fed. The cutter operated at a speed of approximately 3 revolutions per second, with both the feed and pressure set manually. To achieve a high-quality lower edge on the samples and to ensure the durability of the cutting tool, a polytetrafluoroethylene (PTFE) pad was placed underneath each sample. Flat samples, referred to as “dog-bone shape,” were cut using a guiding template for the blade. Standard microtome blades, typically used for preparing histopathological specimens, were employed for this process. The template was composed of two plates that matched the contour of the sample’s geometry. An elastomer material was placed between these plates, and the blade was used to make the cuts. This approach ensured consistent sample geometry, despite the material’s tendency to deform significantly under the pressure of the blade. By using the template, the intended shape was maintained, as the geometry was determined solely by the path of the tool guide, regardless of any deformation in the material.

### 2.2. Sample Dimensions

**Figure A1:**
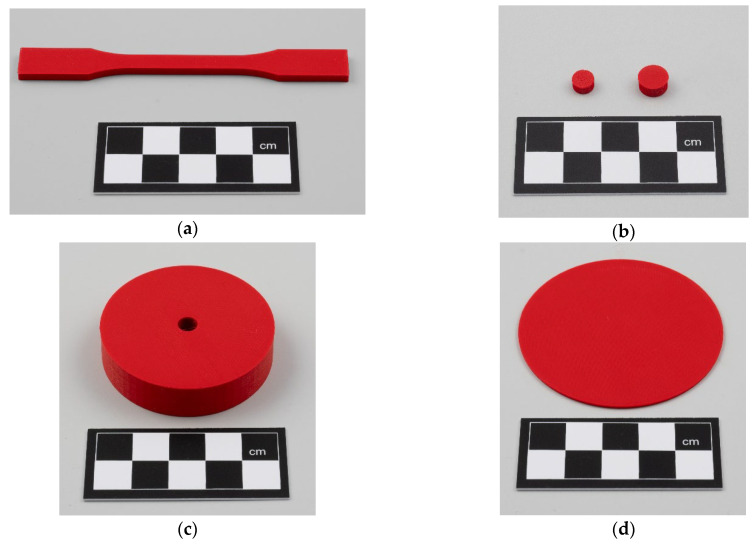
Printed samples for different tests to estimate mechanical properties and sorption and solubility: (**a**) tensile test, (**b**) dynamic testing—split Hopkinson pressure bar, (**c**) abrasion resistance, (**d**) sorption and solubility.

Samples for the tensile strength test were dog-bone shaped (Table A1, Figure A1). Samples for abrasion resistance were cylindrical, with a diameter of 50 mm and a thickness of 10 mm. Samples for the drop-ball test were cylindrical with a diameter of 50 mm and a thickness of 3 mm. Samples for absorption and solubility were prepared according to the ASTM D570 standard [34], which specifies cylindrical specimens with a diameter of 60 mm and a thickness of 1.5 mm (Table A1, Figure A1). The ASTM D570 standard, originally developed for plastic materials, was chosen because there is currently no dedicated standard for water absorption testing of 3D-printed thermoplastic materials used in mouthguard manufacturing. Using this standard enabled comparison with prior studies examining polymeric materials in mouthguard research [35,36]. Dog bone-shape samples and cylindrical samples used as reference material for the tensile strength test, the dynamic test (Split Hopkinson Pressure Bar), and absorption and solubility studies were cut (shape and dimensions were with printed samples) out of 3 mm-thick EVA plates (Erkoflex, Erkodent, Pfalzgrafenweiler, Germany), and samples for absorption and solubility were cut out of 1.5 mm-thick plates (Erkoflex, Erkodent, Pfalzgrafenweiler, Germany). Despite different methods of preparation, all dimensions of material samples—those printed and the EVA control group—had the same dimensions for each conducted test.

### 2.3. Tensile and Compression Strength

The test was performed according to the PN-EN ISO 527:1998 standard [37]. A total of 6 measurements were made for each material. Dog-bone samples were used for this study (Figure A1). A Zwick/Roell Z020 universal testing machine (Zwick/Roell, Ulm, Germany) was used for the tests. The tensile strength test speed was 5 mm/min. Young’s modulus was calculated by software from the strain–stress curve. The strain was calculated by utilising the machine cross-head motion. Additionally, quasi-static compression tests were carried out at room temperature (RT) and 50 °C using an MTS Criterion 45 testing machine equipped with a climate chamber. All quasi-static compression experiments were conducted at a strain rate of 0.001 s^−1^ on samples with a diameter of 8 mm and a height of 4 mm or a diameter of 6 mm and a height of 3 mm, depending on the material. At least three measurements were taken for each type of material.

### 2.4. Split Hopkinson Pressure Bar (SHPB)

**Figure A2:**
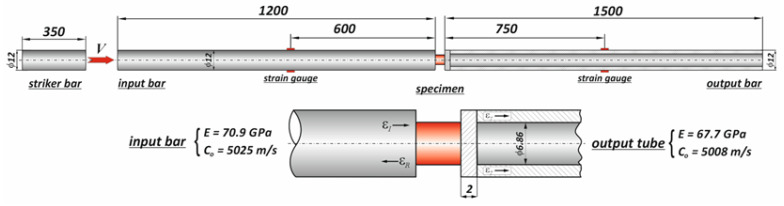
Split Hopkinson pressure bar (SHPB) set-up.

Polymer materials, from the mechanical point of view, belong to the group of materials called soft materials or low-mechanical-impedance materials [38,39,40]. Therefore, the study of soft materials under split Hopkinson pressure bar (SHPB) conditions sometimes requires the use of specialised bar system solutions. In the present work, a modified SHPB set-up equipped with a hollow output bar featuring an end cap instead of a traditional solid output bar was used, as shown in Figure A2. For each type of material, at least three measurements were taken under the same conditions. The total length of the SHPB device, which consists of a gas gun system with a striker bar and two long bars, is about 4.5 m. The 350 mm-long cylindrical striker bar and 1200 mm-long input bar with a standard diameter of 12 mm are made of Al 7075-T6 (Young’s modulus *E* = 70.9 GPa; elastic wave velocity *C*_0_ = 5025 m/s). In turn, as an output bar, a 12 mm hollow bar (tube) with a length of 1500 mm and an inner diameter of 6.86 mm is made of Al 6063-T53 alloy (*E* = 67.7 GPa, *C*_0_ = 5008 m/s) [41,42]. To support the specimen between the bar ends correctly, the end cap was mounted on the front end of the hollow output bar. Applying a hollow bar as the output bar was required due to the problem of too small amplitude of the transmitted wave signal (*ε_T_*), which is a consequence of a significant mismatch of mechanical impedance between the specimen and the metallic bars. The wave signals in the input and output bars were captured using a pair of strain gauges (CEA-13-062UW-350, Vishay Micro Measurements, Wendell, NC, USA) attached symmetrically to the opposite surfaces of the bars and at their midpoints. Typical resistance strain gauges with a gauge length of 1.6 mm were used. The amplified signals from the strain gauges were recorded using a data acquisition system with a frequency band of 1 MHz (SGA-0B V5 unit, ESA Messtechnik, Olching, Germany, and a LeCroy WJ354A oscilloscope, Teledyne LeCroy, Chestnut Ridge, NY, USA).

To minimise wave dispersion (Pochhammer–Chree high-frequency oscillations) and facilitate stress equilibrium, a pulse-shaping technique was employed. Pulse-shaping allows for controlling the time history of the loading pulse [38,43,44]. It was found that for the given SHPB test conditions, the nylon pulse shaper, with a diameter of 3 mm and a thickness of approximately 0.3 mm, ensured the damping of high-frequency oscillations and improved the dynamic stress-state equilibrium in the specimens by impacting striker velocity V. The impact striker bar velocity V applied during all SHPB experiments was the same—18.5 ± 0.3 m/s. Cosmetic Vaseline was applied to the interfaces between the specimens and the bars to minimise ss interfacial friction. The stress–strain curve profiles were determined in the classical way, i.e., in accordance with Kolsky’s theory based on the wave signals ((incident (ε*_I_*), reflected (*ε_R_*), and transmitted (*ε_T_*)) recorded by strain gauges.

### 2.5. Drop-Ball Test

**Figure A3:**
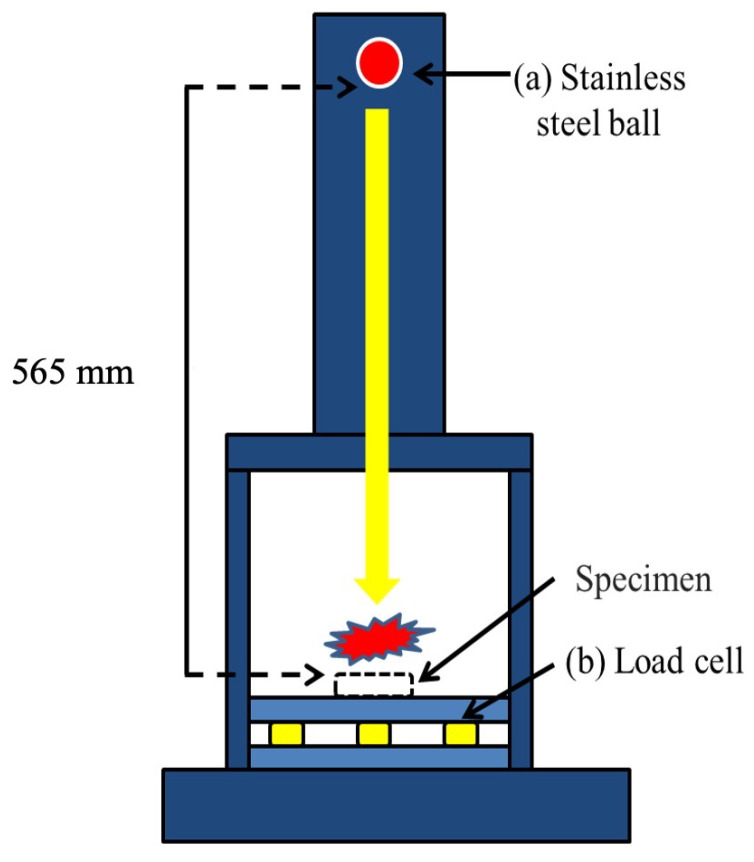
Drop-ball test set-up.

A free-falling stainless-steel ball (weight: 32.6 g, diameter: 20.0 mm), a 10 mm-thick steel platform, and a stainless-steel base plate (180 × 180 × 10 mm) in a modified IM-201 impact testing machine (Tester Sangyo Co., Miyoshi, Japan) were used in the test (Figure A3) with reference to previously published test reports on conventional materials for mouthguards [45]. The ball was released from 600 mm above the specimen, and the impact force generated was measured using a load cell sensor system. The control load without the specimen was set to 723 N. Three dynamic LMB-A-2KN compression load cells with a rated capacity of 2 kN (Kyowa Electronics Instruments Co., Chofu, Japan) were positioned 120° apart on the base plate of the impact tester. The stainless-steel platform was then placed on top of the load cells. The change in force during the impact test was recorded with the three load cells, and the sum of the measured force values was calculated. After impact, the maximum of the sum of the measured forces was taken as the maximum impact force (MIF). Maximum impact force is a critical parameter in evaluating the protective performance of intra-oral mouthguards. Lower MIF values indicate better energy dissipation by the material, reducing the force transmitted to oral cavity structures during the impact.

### 2.6. Abrasion Resistance

The abrasion resistance of the examined materials was assessed using a Three-Media Abrasion System (SD-Mechatronik, Feldkirchen-Westerham, Germany). Prior to testing, each sample was weighed using a precision analytical balance (model XA/82/220/X, RADWAG, Radom, Poland) to establish its initial mass. Subsequently, the specimens were mounted in the abrasion device, where the test material was configured as a rotating disc. During the procedure, the sample disc was set to rotate at a speed of 60 revolutions per minute (rpm) in the opposite direction to a counter-sample made of steel, which rotated at 130 rpm. The test parameters were selected based on the manufacturer’s instructions. A constant normal force of 10 N was applied, pressing the steel antagonist wheel against the test specimen to simulate abrasive interaction under controlled conditions. After 200,000 revolutions of the test material’s wheel, the samples were cleaned and weighed. The weight loss from before to after the abrasion test was calculated (*n* = 3/group).

Additional control samples were prepared from thermoformed EVA (Erkoflex), thermoformed on a metal cylinder fabricated explicitly for the tested machine, with additional cuts on its surface to enhance the hold. However, due to the forming method, it was not possible to produce specimens at least 5 mm thick that were uniform in geometry and surface. Several techniques were employed to standardize the outer surface; however, all methods proved insufficient. This resulted in inconsistent surface contact with the abrasion wheel. This led to uneven wear and sample deformation at random points. As a result, the test could not be performed under the same conditions, and Braskem BIO EVA remained the only reference material for the abrasion test.

### 2.7. Absorption and Solubility

The test design was conducted in accordance with the ASTM D570 standard. Five cylindrical specimens were prepared for each tested material. The samples were conditioned for 24 h at 50 °C, and after cooling, they were weighed (dry weight—m_d_). Next, the tested samples were put into distilled water at room temperature. The samples were removed from the water after one month, and all surface water was wiped off with a dry cloth and weighed (sorbed weight—m_s_). After one month, samples were reconditioned for 24 h at 50 °C, and after cooling, the specimens were weighed (reconditioned weight—mr). Absorption (Ab) and solubility (Ds) were calculated according to Equations (1) and (2).

(1) $$Ab\left[\%\right]=\frac{m_{s}\cdot m_{d}}{m_{d}}\cdot 100\%$$

(2) $$Ds\left[\%\right]=\frac{m_{d}-m_{r}}{m_{d}}\cdot 100\%$$

### 2.8. Statistical Analysis

The study’s results were subjected to statistical analysis. The values of the analysed variables are presented using means, standard deviation, and minimum and maximum values. The normality of the distribution of the variables was checked using the Shapiro–Wilk test. Evaluation of differences between groups for tensile strength, abrasion, absorption, and solubility was performed using ANOVA along with Scheffés post hoc test. This test was selected due to its conservative nature, which controls for type I error when making multiple comparisons. The analysis was performed using PQStat (version 1.8.6.) and Statistica 9.1 software. For drop-ball test results, ANOVA followed by the Tukey–Kramer test was conducted. This test was chosen for its suitability in handling unequal sample sizes while controlling for type I error. A significance level of *p* < 0.05 was adopted to indicate the existence of statistically significant differences or relationships.

## 3. Results

### 3.1. Tensile Strength

The highest ultimate tensile strength (UTS) was measured for Copper 3D PLActive and Spectrum Medical ABS (Table 2). DSM Arnitel ID 2045 had slightly lower UTS than 3D-printed EVA. During tensile strength testing, the NinjaFlex and EVA Erkoflex samples did not reach the point of rupture due to their high elasticity. Therefore, the tensile strength values presented for these materials correspond to the stress measured at 440% elongation, based on the displacement of the testing machine’s base. Although these values do not represent the ultimate tensile strength, they are included in the comparative analysis (Table 2) and are marked with an asterisk for clarity. The notations “a,” “b,” etc. in all tables, mean that a given material yielded a similar result (not statistically significantly different) from material in group “a.” The letter designations denote “homogeneous” materials. The earlier the letter in the alphabet, the lower the value.

A comparison was also made of the rapture tensile strength of the tested samples (Table 3). As mentioned above, NinjaFlex and EVA Erkopress were excluded from this evaluation. The comparison of Young’s moduli is presented in Table 4.

### 3.2. Split Hopkinson Pressure Bar

In this research, nominal stress–strain curves were used in conjunction with true stress–strain curves due to the complexities introduced by the additive manufacturing process—additively manufactured materials are highly sensitive to parameters like layer thickness, temperature gradients, and cooling rates—and the nature of the materials involved. Nominal stress and strain provide a simplified framework that aligns better with the mechanical behaviour observed in these materials. As the literature reports, additive manufacturing (AM) often results in residual stresses due to thermal gradients and material extrusion processes. These stresses can lead to warpage and layer delamination, complicating the interpretation of true stress–strain data [46]. Moreover, the layer-by-layer construction of additive manufactured parts results in anisotropic properties, where nominal stress–strain measurements can more effectively represent the overall mechanical behaviour than true values, which assume uniformity [47]. Anisotropy in additively manufactured polymers means their properties vary with the direction they are measured in. This directional dependence is the result of the layer-by-layer printing process, mostly resulting in weaker bonds between layers and different properties along the build (Z) axis compared to the X and Y axes. Additionally, the behaviour of polymers under load can be highly nonlinear, especially at large strains. Nominal measures provide a more straightforward approach to understanding these behaviours without the complications of true stress–strain calculations, which can become impractical [48,49,50].

**Figure 1:**
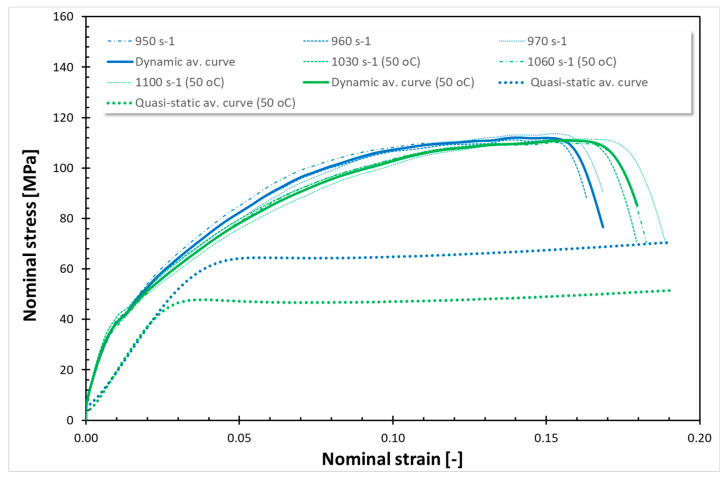
Stress–strain curves for Spectrum Medical ABS.

**Figure 2:**
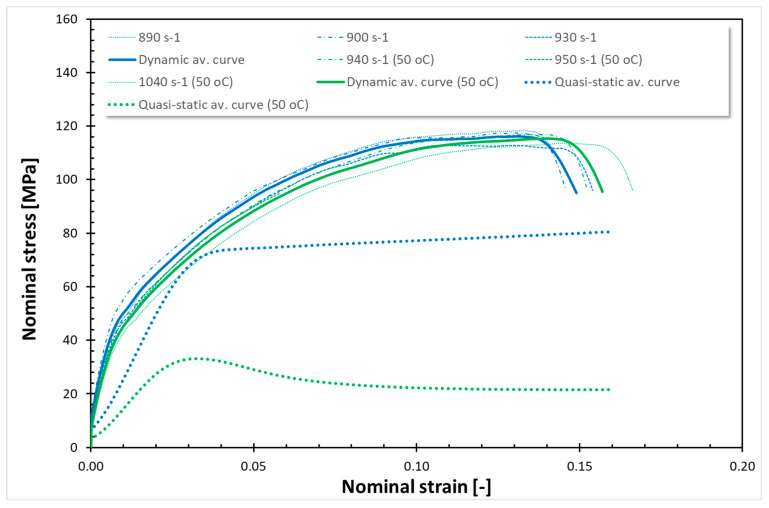
Stress–strain curves for Copper PLActive.

**Figure 3:**
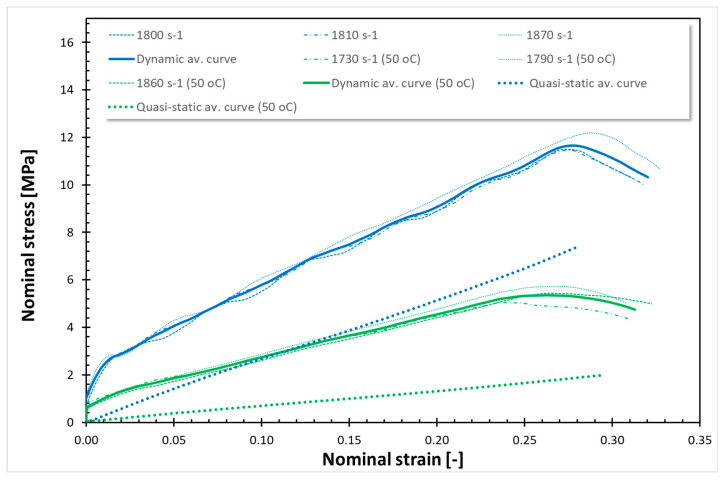
Stress–strain curves for EVA Erkoflex (solid).

**Figure 4:**
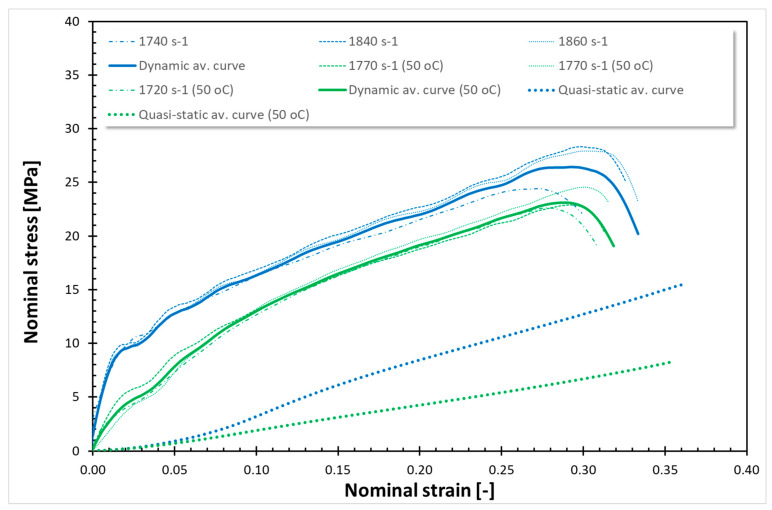
Stress–strain curves for Braskem BIO EVA (3D-printed).

**Figure 5:**
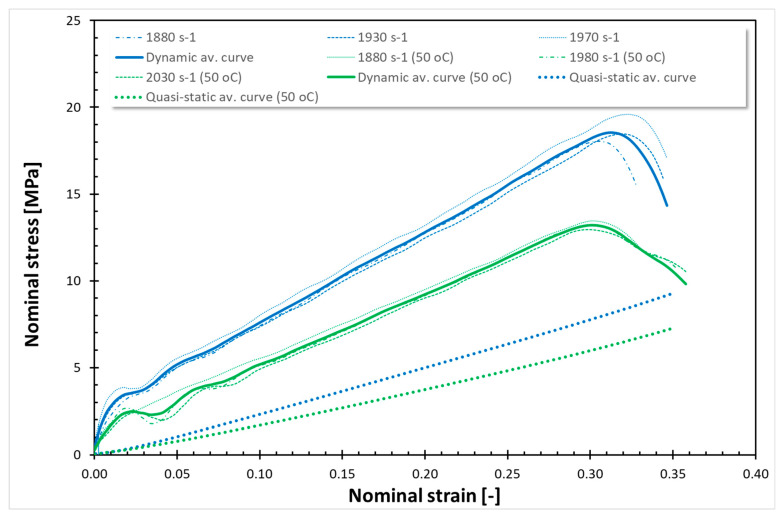
Stress–strain curves for NinjaFlex (TPU).

**Figure 6:**
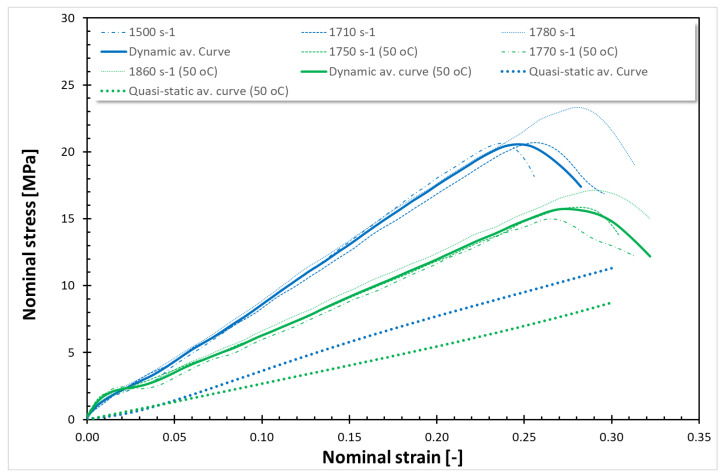
Stress–strain curves for DSM Arnitel ID 2045.

The findings from all conducted tests, encompassing both quasi-static and dynamic compression at room temperature (RT) and 50 °C, are compiled in Figure 1, Figure 2, Figure 3, Figure 4, Figure 5 and Figure 6. Furthermore, Table 5 presents the stress values at specific strains of 0.05, 0.10, and 0.15 for quasi-static loading, as well as at strains of 0.05, 0.15, and 0.25 for dynamic loading, along with the compressive moduli derived from the nominal stress–strain curves. In the analysis of the results, it is imperative to highlight that Spectrum Medical ABS and Copper PLActive materials are classified as thermoplastics, distinguishable by their amorphous or semi-amorphous structures. In contrast, Braskem BIO EVA, NinjaFlex (TPU), and DSM Arnitel ID 2045 are representative of thermoplastic elastomers, which integrate the elastic characteristics of rubbers with the advantageous processing properties of plastics. Moreover, it is crucial to recognise that the quasi-static tests operate under isothermal conditions, whereas dynamic loading subjects the specimens to adiabatic heating, potentially leading to significant temperature elevations. As elevated temperatures can alter the mechanical properties of materials, it is essential to account for the implications of adiabatic heating on the compressive behaviour of these materials.

Analysing the curves for the static compression test for thermoplastic polymers (Figure 1 and Figure 3), typical nonlinear viscoelastic behaviour followed by yield and plastic flow commencing at approximately ~4% nominal strain for Spectrum Medical ABS and Copper PLActive at RT (~3% at 50 °C) is observed. Static curves for all those cases exhibit almost the same behaviour before passing the yield point. However, the yield point varies slightly for these materials, with the highest stress values (yield point) for Copper PLActive (~75 MPa). Once the yield point is exceeded, the stress–strain behaviour changes to plastic deformation. During plastic deformation, Spectrum Medical ABS (RT and 50 °C) and Copper PLActive (only RT) develop a relatively constant plastic flow. However, Copper PLActive (only at 50 °C) presents a prominent post-peak strain softening. The compressive properties of thermoplastic polymers are greatly affected by temperature. The observed yield point at 50 °C is about 15 MPa lower for Spectrum Medical ABS, whereas for Copper PLActive, it is 40 MPa lower than that at RT. There are also visible differences in the compressive moduli. The compressive modulus of Spectrum Medical ABS remains stable at room temperature and 50 °C, in contrast to Copper PLActive, which shows a decreasing elastic modulus with rising temperature. This trend is consistent with that of the literature [49].

As shown in Figure 1 and Figure 2, the dynamic stress–strain curves for thermoplastics under high strain rates exhibit a marked difference. During dynamic compression, adiabatic heating associated with large inelastic deformation occurs in the specimen. Note that the shapes of the dynamic stress–strain curves are different from those of the quasi-static stress–strain curves. The dynamic stress–strain curves demonstrate an initial, nearly linear response, which transitions to a nonlinear region before exhibiting strain-hardening behaviour. The dynamic compressive strength of Spectrum Medical ABS and Copper PLActive is significantly higher than their static compressive strength. The dynamic compressive strength for Spectrum Medical ABS and PLA is almost the same at RT and 50 °C. It is worth noting that the nature of curves is the same for those thermoplastic materials for quasi-static compression at RT and 50 °C, as well as for dynamic compression at RT and 50 °C. For quasi-static (QS) loading, a decrease of 25% and 60% of σ_0.05_, σ_0.10_, and σ_0.15_ at 50 °C was observed for Spectrum Medical ABS and Copper PLActive, respectively. For dynamic testing, a decrease in stresses (σ0.05, σ0.10, and σ0.15) was also noticed at 50 °C and was ~5%, and ~5% for Spectrum Medical ABS and Copper PLActive, respectively.

Figure 3, Figure 4, Figure 5 and Figure 6 show the quasi-static compressive and dynamic stress–strain curves for 3D-printed thermoplastic elastomers at RT and 50 °C (for EVA; Erkoflex results for the solid material have been added as a reference). It is worth noting that under conditions of constant temperature and short-term loading, elastomers typically demonstrate isotropic and incompressible properties. Through QS compression until 30–40% strain (depending on the material), Braskem BIO EVA, NinjaFlex (TPU), and DSM Arnitel ID 2045 exhibit a nonlinear hyperelastic deformation without observable yielding behaviour. For those materials, static loading induces negligible changes in molecular chain orientation or waviness, resulting in full material recovery and the absence of permanent deformation. Consequently, the conventional 0.2% strain criterion for yield stress is inapplicable. For the tested 3D-printed elastomers under static loading, the yield stress is defined as 0 MPa. Nevertheless, even a slight temperature increase affects Young’s modulus. In all cases, a higher Young’s modulus was obtained for tests conducted at 50 °C. EVA solid/plate exhibited the most significant change in Young’s modulus.

The dynamic stress–strain response of thermoplastic elastomers (Braskem BIO EVA, NinjaFlex TPU, and DSM Arnitel ID 2045) is characterised by a nonlinear viscoelastic region, transitioning to a distributed yield point. Subsequent deformation is unrecoverable, indicating viscoplastic flow before stiffening at higher strain levels. High-rate compressive tests reveal that the tested material exhibits softening after exceeding the dynamic yield point, which is especially noticeable for NinjaFlex TPU. However, the increased loading rate under impact conditions elevates internal friction between molecular chains, requiring greater energy for segmental motion. This results in improved mechanical strength. As in the case of previous tests, a significant decrease in strength properties was observed in tests conducted at a temperature of 50 °C. Furthermore, a reduction in compressive moduli was also noticed.

For the tested thermoplastic elastomers, the nature of curves, both for static at RT and 50 °C, as well as for dynamic compression at RT and 50 °C, is similar. Nevertheless, the decrease in stresses at 50 °C is not uniform, unlike the case of the tested thermoplastic materials. It is visible at elevated temperature that as the strain increases, the stress decreases more: for QS compression of Braskem BIO EVA (3D-printed), there is a decrease in σ_0.05_, σ_0.15_, and σ_0.25_ of ~0%, ~50%; and ~30%, respectively; for NinjaFlex (TPU) ~2%, ~25%, and ~25%; and for DSM Arnitel ID 2045 ~0%, ~30%, and ~30%. For dynamic compression, the decrease in σ_0.05_, σ_0.15_, and σ_0.25_ for Braskem BIO EVA was ~40%, ~10%, and ~14%, respectively; for NinjaFlex (TPU) ~45%, ~30%, and ~28%; for DSM Arnitel ID 2045 ~20%, ~30%, and ~30%. For QS compression of EVA Erkoflex (solid), there was a decrease in stress of ~97% at strain 0.05 and ~75% and ~75% at strains 0.15 and 0.25, respectively, while for the dynamic testing, decreases in stress of ~95% at strain 0.05 and of ~50% and ~50% at strains 0.15 and 0.25 were observed.

### 3.3. Drop-Ball Test

Rigid materials, such as Copper PLActive or Spectrum Medical ABS, transmitted the highest force during the test. The highest absorption was observed for DSM Arnitel ID 2045 (Table 6). The differences between Erkoflex material and 3D-printed Braskem BIO EVA were not statistically significant.

### 3.4. Abrasion Resistance

Samples 3D printed from NinjaFlex filament had the highest abrasion loss (Table 7). DSM Arnitel 2045 was not resistant to abrasion either, with a mean result of 1341. The most resistant were the more rigid materials Copper 3D PLActive and Spectrum Medical ABS, and the difference between those two was not statistically significant.

### 3.5. Absorption and Solubility

The highest absorption was achieved by samples 3D-printed with NinjaFlex filament, both after one week of evaluation (Table 8). The 3D-printed EVA material showed the highest diversity of absorption among the tested materials. The absorption, similar to that achieved by EVA used for mouthguard thermoforming, had DSM Arnitel ID 2045. After one month of evaluation, the absorption of this material was higher than that of EVA Erkoflex, but those results were not statistically significant. The samples of Braskem Bio EVA had the highest standard deviation, which might mean that they are not homogeneous.

The highest solubility of tested materials was noted for NinjaFlex. The differences between other tested samples were not statistically significant (Table 9).

## 4. Discussion

The use of filament 3D-printed materials enables the precise and cost-effective fabrication of intra-oral appliances [28,29]. Among their advantages for mouthguard manufacturing are easy personalisation, rapid prototyping, and the possibility of easy reproduction [51]. Our research compared mechanical properties of five various filaments with different characteristics—both elastic and stiff options. The strength of this research was the inclusion of ethylene vinyl acetate (EVA) as a control group to compare the results with the current gold standard in mouthguard fabrication. Our study showed that the most favourable material for mouthguard manufacturing was the biocompatible and non-toxic thermoplastic copolymer (TPE)-DSM Arnitel ID 2045. Its application in mouthguards manufacturing was previously explored by Saunders et al. [52]. They compared various designs of 3D-printed samples, and found that the properties of this filament material were superior to those of EVA [52]. They showed that it dissipated 25% more energy than ethylene vinyl acetate at high and medium strain rates. Our current study also demonstrated that during a drop-ball test—an established method for evaluating mouthguard materials—significantly more energy was dissipated than EVA. DSM Arnitel ID 2045 also exhibited low absorption and solubility, confirming its suitability for use in the oral cavity. NinjaFlex is a thermoplastic polyurethane that has been widely used in medical applications [53,54,55,56]. However, due to high absorption, solubility, abrasion loss, and lower ability to absorb impact force, it is an unsuitable choice for intra-oral protective splints. The protective properties of the 3D-printed EVA were similar to samples from solid plates in a drop-ball test. However, absorption and solubility tests resulted in a significant range in results, which may have been caused by low homogeneity or repeatability of samples. Additionally, Braskem BIO EVA was not been tested for biocompatibility. Its further application would require a safety test and additional precautions. In the current research, two rigid, biocompatible materials—Spectrum Medical ABS and Copper PLActive—were also included. Due to their properties, they cannot be considered for a one-material protective splint, but they could be further tested in combination with DSM Arnitel ID 2045 as a hard insert in multi-material construction. This design has been discussed in the literature by several authors [24,57,58,59]. The inclusion of a hard insert may result in a 50% reduction in the impact strength measure at the incisors [27]. Due to the low abrasion resistance of DSM Arnitel 2045, the use of a rigid layer on the occlusal surface could also be suitable for patients with bruxism. Tribst et al. [60] described a mouthguard that incorporates a rigid material for patients experiencing increased masticatory forces.

There are various methods for testing the mechanical properties of materials suitable for dental applications [44,51,61]. Evaluation of elastic biocompatible materials in our previous study showed that resins dedicated to indirect bonding can be considered for mouthguard application. The best properties were achieved by Keyortho IBT [8]. Nasrollahzadeh et al. [27] also showed that even a single layer of 3 mm Keyortho IBT resin performed better than a mouthguard laminated with two EVA and one SBS sheets. In the current study, the highest tensile strength was achieved by rigid materials—Copper 3D PLActive and Spectrum Medical ABS. NinjaFlex and Erkoflex (EVA dedicated for thermoforming) did not rupture during testing due to their high elasticity. The tensile strength of these materials was measured at 440% elongation. DSM Arnitel ID 2045 exhibited higher tensile strength values than solid EVA (Erkoflex). It had a lower rupture point than 3D-printed EVA, but there were no statistically significant differences in Young’s modulus.

In the current study, two methods of impact absorption test—the drop-ball test and split Hopkinson pressure bar—were employed. In both, the most favourable properties were for DSM Arnitel ID 2045. Drop-ball tests were previously conducted on various types of mouthguard materials, including 3D-printed mouthguards [9,62,63,64,65]. Arfi et al. [25] showed that 3D-printed splints have slightly better shock absorption abilities that thermoformed “boil and bite” splints, with mean deceleration of 277–302 g. The authors also pointed out that the lowest thickness variation was for appliances made with additive manufacturing. Huang et al. [66] used a drop-ball test to evaluate the properties of innovative shear-stiffening mouthguards (SSMs). These showed high shock absorption abilities, with about 60% reduction in peak force compared to commercial EVA (Erkoflex and Erkoloc-pro). Further modifications and tests on materials for 3D printing may bring us closer to highly protective, comfortable mouthguards with predictable dimensions. The results of the split Hopkinson pressure bar (SHPB) test provide critical insights into the dynamic mechanical behaviour of materials under high strain rates. It can simulate impact conditions encountered during mouthguard use. The tests revealed that thermoplastic materials such as Spectrum Medical ABS and Copper3D PLActive exhibited a different response compared to thermoplastic elastomers (Figure 1, Figure 2, Figure 3, Figure 4, Figure 5 and Figure 6). These materials demonstrated significantly higher stress values under both quasi-static and dynamic loading conditions. Notably, under elevated temperature conditions, these thermoplastics showed greater stability in their mechanical response during dynamic testing. It should be mentioned that the mechanical properties of additively manufactured polymers are significantly affected by environmental temperature, primarily due to their viscoelastic nature. Elevated temperatures lead to a reduction in both tensile strength and Young’s modulus because they weaken the intermolecular forces within the polymer. This weakening makes the material less resistant to deformation and fracture. Some polymers may exhibit a transition from brittle to ductile behaviour as temperature increases. Those characteristics again suggest their potential application as reinforcing components in multi-material mouthguard designs, where they could serve to absorb and distribute impact energy across varying environmental conditions. Thermoplastic elastomers—including DSM Arnitel ID 2045, NinjaFlex TPU, and Braskem BIO EVA—exhibited generally higher mechanical performance than the control material EVA Erkoflex. These materials also demonstrated superior stability at elevated temperatures and under dynamic loads, reinforcing their suitability as primary base materials in energy-absorbing protective devices. DSM Arnitel ID 2045 was also tested using a split Hopkinson pressure bar by Saunders et al. [52], who showed that it dissipated 25% more energy than EVA in high-rate loading tests. The authors also noted different behaviour of printed samples from EVA when subjected to force, as they tended to fragment.

Mouthguards are used in demanding environments: not only do they absorb impact forces, but they are also subjected to the influence of saliva and occlusal loads from opposing teeth. In addition to evaluating the biocompatibility and biosafety of materials, it is important to consider the oral environment, which significantly affects the performance of materials used in this setting. Previous research has also shown that elastic materials used in mouthguards undergo significant changes after usage or hygienic procedures [67,68,69]. Parameters that can be indirectly used for the preliminary assessment of material stability are absorption and solubility. Increased values of these parameters suggest that the material may absorb large amounts of solvents, which can lead to hydrolysis and degradation. High solubility values indicate that the material contains unstable components that are leached into the aqueous environment [70,71,72]. The high absorption and solubility demonstrated by NinjaFlex in this study render the material unsuitable for intra-oral application. The high variability in the results of sorption and solubility of Braskem BIO EVA may be attributed to its method of production. According to the manufacturer, it is made of sugarcane—a biobased and recyclable alternative to fossil-based EVA. Unfortunately, the material’s heterogeneity represents a significant drawback that prevents the use of this particular filament for mouthguard fabrication. Current recommendations suggest that the water sorption of mouthguard material should be no greater than 0.5 wt.% [73]. DSM Arnitel ID 2045 exhibited similar sorption and solubility properties to the currently used mouthguard material, EVA, both meeting the requirements. Another important parameter for materials intended for dental mouthguards is abrasion resistance. In the present study, it was not possible to perform abrasion tests on the thermoformable material (EVA Erkoflex). During preliminary polishing procedures, this material exhibited significant wear. As this is the currently most used material for mouthguards, slightly higher abrasion values observed for DSM Arnitel ID 2045 should not raise major concerns in this context. Considering the numerous advantages of this material, it is necessary to further verify the frequency of replacement of mouthguards made from such material. It should also be evaluated whether under functional loading, the protective splint would be susceptible to damage through the mechanisms described by Saunders et al. [52]. However, currently, most mouthguard users do not replace them often enough. That considered, the visible change in the protective splint used occurring after damage may not necessarily be a drawback of such an appliance, but a clear message for its user that it should be replaced.

This study is the first to compare different mechanical characteristics of filament 3D-printed materials that could be used for the additive manufacturing of mouthguards. The strong point of this research is that various tests were conducted to provide a full comparison of material samples. Additionally, the inclusion of ethylene vinyl acetate as a reference material enabled the comparison of commonly used solutions in mouthguard fabrication. However, there are some limitations of this study that should be listed. The comparison of material samples is a simplified method of estimating the protective characteristics of the final appliance: comparison of properties made on full splints might provide slightly different results. The high elasticity of NinjaFlex and EVA Erkoflex also necessitated the tensile strength for these materials being measured at 440% elongation. Material extrusion (MEX) of tested elastic materials had its challenges—to achieve a smooth surface of prints, we needed to adjust print parameters. The choice of different parameters could have influenced the results obtained, and should be considered when referring to such results. To avoid any mistakes, we have listed all changes that were necessary during sample printing. Future work should also focus on investigating complex configurations that combine rigid and elastic components. Evaluating and optimising the mechanical synergy between these material types under impact conditions will be crucial for the development of next-generation protective oral devices.

## 5. Conclusions

In the present study, we compared the physico-mechanical properties of commercially available filament materials for additive manufacturing in terms of their potential use in the fabrication of custom mouthguards. The conducted evaluation included tensile strength, split Hopkinson pressure bar, drop-ball impact, abrasion resistance, absorption, and solubility tests. Among the tested filaments, DSM Arnitel ID 2045 is the best choice for a single-material custom mouthguard. In a drop-ball test, it transmitted the lowest force among the test materials of 376,4 N, while the control samples of EVA materials transmitted 411,5 N. Although it exhibited low abrasion resistance, it did not have statistically different absorption or solubility from the control material, which makes it suitable for intra-oral application in mouthguard fabrication.

## Figures and Tables

**Table 1:** Material extrusion (MEX) parameters of different filament materials. A—recommended by filament manufacturer; B—used in the current study; ND—no data.

Material	Copper PLActive		Spectrum Medical ABS		Braskem Bio EVA		DSM Arnitel ID 2045		NinjaFlex (TPU)	
Parameters	A	B	A	B	A	B	A	B	A	B
Nozzle/print temperature—1st layer [°C]	190–210	205	235–255	250	220–250	230	220–245	230	225–250	245
Nozzle/print temperature—subsequently layers [°C]	190–210	200	235–255	245	220–250	230	220–245	230	225–250	240
Bed temperature [°C]	0–60	50	100	110	20–40	30	40–60	80	50	50
Fan—first two layers [%]	ND	0	0–25	0	50–100	0	0	0	0	0
Fan—subsequently layers [%]	ND	100	0–25	15	50–100	0	0	0	ND	0
Layer thickness—1st layer [mm]	0.1	0.15	0.05–0.30	0.15	ND	0.15	>0.1	0.1	ND	0.15
Layer thickness—subsequently layers [mm]	0.1	0.1	0.05–0.30	0.1	ND	0.1	>0.1	0.15	ND	0.1
Print speed [mm/s]—infill|contour	40–50	80|45	30–150	80|45	20–40	40|30	ND	40|40	15–35	30|30

**Table 2:** Statistical analysis of ultimate tensile strength results.

	N	Mean	SD	Min	Max
Copper 3D PLActive	6	48.55 f	1.13	47.20	50.40
ABS Medical Spectrum	6	45.38 e	0.44	44.80	45.90
DSM Arnitel ID 2045	6	11.47 b	1.14	9.29	12.60
Braskem BIO EVA	6	16.28 d	1.05	14.40	17.10
NinjaFlex *	6	14.60 c	0.69	13.40	15.30
EVA Erkoflex *	5	6.95 a	0.43	6.45	7.59
F = 2488.110, *p* < 0.001					

* Tensile strength value corresponds to the stress at 440% elongation. The material did not reach the point of rupture during testing.

**Table 3:** Statistical analysis of tensile strength results—rupture of tested materials.

	N	Mean	SD	Min	Max
Copper 3D PLActive	6	48.55 d	1.13	47.20	50.40
ABS Medical Spectrum	6	34.93 c	0.58	34.10	35.80
DSM Arnitel ID 2045	6	10.88 a	1.00	8.96	11.80
Braskem BIO EVA	6	15.50 b	1.19	14.40	17.10
F = 1829.265, *p* < 0.001					

**Table 4:** Statistical analysis of Young’s moduli obtained during the tensile strength test.

	N	Mean	SD	Min	Max
Copper 3D PLActive	6	1711.67 b	119.57	1600.00	1920.00
ABS Medical Spectrum	6	1578.33 b	142.89	1450.00	1780.00
DSM Arnitel ID 2045	6	50.60 a	8.06	38.40	59.20
Braskem BIO EVA	6	100.93 a	3.30	97.60	106.00
NinjaFlex	6	27.40 a	1.98	24.10	29.40
EVA Erkoflex	5	21.62 a	2.44	18.80	25.50
F = 670.133, *p* < 0.001					

**Table 5:** Measured mechanical properties (true stress—true strain).

	Quasistatic Testing								Dynamic Testing							
	σ_0.05_ [MPA]		σ_0.10_ [MPA]		σ_0.15_ [MPA]		Compressive Modulus [GPa]		σ_0.05_ [MPA]		σ_0.10_ [MPA]		σ_0.15_ [MPA]		Compressive Modulus [GPa]	
	RT	50 °C	RT	50 °C	RT	50 °C	RT	50 °C	RT	50 °C	RT	50 °C	RT	50 °C	RT	50 °C
Spectrum medical ABS	64	47	64	47	68	49	1.5	1.5	83	79	107	102	112	111	1.3	1.2
Copper PLActive	74	29	77	22	80	22	1.9	1.2	90	85	113	110	115	115	1.3	1.3
	**σ_0.05_ [MPA]**		**σ_0.10_ [MPA]**		**σ_0.15_ [MPA]**		**Compressive Modulus [MPa]**		**σ_0.05_ [MPA]**		**σ_0.10_ [MPA]**		**σ_0.15_ [MPA]**		**Compressive Modulus [MPa]**	
	**RT**	**50 °C**	**RT**	**50 °C**	**RT**	**50 °C**	**RT**	**50 °C**	**RT**	**50 °C**	**RT**	**50 °C**	**RT**	**50 °C**	**RT**	**50 °C**
EVA Erkoflex (solid)	1.4	0.4	3.9	1.0	6.3	1.7	28.5	7.3	4.1	1.9	7.5	3.8	10.5	5.3	50	24
Braskem Bio EVA	0.7	0.7	6.1	3.1	10.5	5.4	18	14	12.8	7.7	19.3	17.4	25.0	21.5	173	132
NinjaFlex (TPE)	0.7	0.6	2.4	1.7	4.3	3.1	13.5	11	2.1	1.6	4.6	3.5	7.1	5.6	27	20
DSM Arnitel ID 2045	1.3	1.3	5.7	4.0	9.4	6.7	28	27	4.3	3.5	13.1	9.3	20.5	14.8	73	49

**Table 6:** Comparison of forces transmitted in the drop-ball test.

	1	2	3	4	5	AVE	S.D	%
Control	708.1	727.7	739.4	696.4	743.0	722.9	20.1	
EVA Erkoflex	410.3	399.8	411.6	415.9	419.7	411.5	7.5	56.9
Copper PLActive	695.7	672.3	686.9	658.3	680.7	678.8	14.3	93.9
DSM Arnitel ID 2045	388.2	386.3	344.2	395.5	367.6	376.4	20.7	52.1
Braskem BIO EVA	430.3	430.5	457.4	439.3	434.9	438.5	11.2	60.7
Spectrum Medical ABS	646.0	676.5	701.9	718.2	703.5	689.2	28.4	95.3
NinjaFlex	405.2	420.2	420.6	413.8	410.6	414.1	6.5	57.3

**Table 7:** Statistical analysis of abrasion resistance of tested materials.

	N	Mean	SD	Min	Max
Copper 3D PLActive	3	0.089 a	0.010	0.081	0.101
ABS Medical Spectrum	3	0.129 a	0.006	0.123	0.134
DSM Arnitel ID 2045	3	1.341 c	0.080	1.263	1.424
Braskem BIO EVA	4	0.568 b	0.118	0.402	0.669
NinjaFlex	3	1.951 d	0.167	1.764	2.085

**Table 8:** Statistical analysis of absorption after one week and one month.

		One Week		One Month	
	N	Mean	SD	Mean	SD
Copper 3D PLActive	5	1.197 ab	0.145	1.563 b	0.243
ABS Medical Spectrum	5	1.015 ab	0.010	1.099 ab	0.028
DSM Arnitel ID 2045	5	0.194 a	0.014	0.203 a	0.018
Braskem BIO EVA	5	1.676 bc	1.242	1.610 b	1.123
NinjaFlex	5	2.700 c	0.354	4.408 c	0.357
EVA Erkoflex	5	0.119 a	0.008	0.27 a	0.014
		F = 15.367 *p* < 0.001		F = 47.240 *p* < 0.001	

**Table 9:** Statistical analysis of solubility after one month.

	N	M	SD	Min	Max
Copper 3D PLActive	5	0.001 a	0.005	−0.005	0.005
ABS Medical Spectrum	5	0.005 a	0.002	0.001	0.006
DSM Arnitel ID 2045	5	−0.002 a	0.013	−0.011	0.013
Braskem BIO EVA	5	0.002 a	0.002	0.000	0.004
NinjaFlex	5	0.347 b	0.009	0.337	0.356
EVA Erkoflex	5	0.007 a	0.015	−0.007	0.026
F = 1237.780, *p* < 0.001					

## Data Availability

The original contributions presented in this study are included in the article. Further inquiries can be directed to the corresponding author.

## Appendix A

**Table A1:** Dimensions of 3D models of samples for different tests to estimate mechanical properties and sorption and solubility of analysed materials.

Tensile Test	Dynamic Testing—Split Hopkinson Pressure Bar
l_T_ = 150 mm l_0_ = 50 mm h = 4 mm d = 10 mm b = 20 mm R = 60 mm	Φ_1_ = 8 mm h_1_ = 4 mm Φ_2_ = 6 mm h_2_ = 3 mm
Abrasion Resistance	Drop-Ball Test
h = 10 mm Φ = 50 mm	h = 3 mm Φ = 50 mm
Sorption and Solubility	
h = 1.5 mm Φ = 60 mm	
